# The COVID-19 pandemic and Alzheimer’s disease: mutual risks and mechanisms

**DOI:** 10.1186/s40035-022-00316-y

**Published:** 2022-09-11

**Authors:** Feng Chen, Yanting Chen, Yongxiang Wang, Qiongwei Ke, Lili Cui

**Affiliations:** 1 Guangdong Key Laboratory of Age-Related Cardiac and Cerebral Diseases, Department of Neurology, Affiliated Hospital of Guangdong Medical University, Zhanjiang, China; 2 Key Laboratory of Animal Models and Human Disease Mechanisms of the Chinese Academy of Sciences & Yunnan Province Kunming Institute of Zoology Chinese Academy of Sciences, Kunming, Yunnan China

**Keywords:** SARS-CoV-2, COVID-19, Alzheimer’s disease, ACE2, APOE, Age, Neuroinflammation

## Abstract

Coronavirus disease 2019 (COVID-19), which is caused by severe acute respiratory syndrome coronavirus 2 (SARS-CoV-2), is a life-threatening disease, especially in elderly individuals and those with comorbidities. The predominant clinical manifestation of COVID-19 is respiratory dysfunction, while neurological presentations are increasingly being recognized. SARS-CoV-2 invades host cells primarily via attachment of the spike protein to the angiotensin-converting enzyme 2 (ACE2) receptor expressed on cell membranes. Patients with Alzheimer’s disease (AD) are more susceptible to SARS-CoV-2 infection and prone to severe clinical outcomes. Recent studies have revealed some common risk factors for AD and COVID-19. An understanding of the association between COVID-19 and AD and the potential related mechanisms may lead to the development of novel approaches to treating both diseases. In the present review, we first summarize the mechanisms by which SARS-CoV-2 invades the central nervous system (CNS) and then discuss the associations and potential shared key factors between COVID-19 and AD, with a focus on the ACE2 receptor, apolipoprotein E (*APOE*) genotype, age, and neuroinflammation.

## Introduction

The outbreak of coronavirus disease 2019 (COVID-19) caused by severe acute respiratory syndrome coronavirus 2 (SARS-CoV-2) has already resulted in more than 540 million infections and over 6 million deaths worldwide as of 27 June 2022 (https://covid19.who.int). The aged individuals and those with comorbidities are at a particularly high risk of poor outcomes [1]. SARS-CoV-2 is a positive-sense, single-stranded RNA virus with four major structural proteins: envelope, membrane, spike (S) and nucleocapsid phosphoprotein [2]. SARS-CoV-2 infects target cells primarily via interaction of the receptor-binding domain of the S protein with the cellular angiotensin-converting enzyme 2 (ACE2) receptor after activation of the S protein by transmembrane serine protease 2 (TMPRSS2) [3]. Thus, the expression and distribution of ACE2 is critical for SARS-CoV-2 infection. The S protein is composed of two functional subunits, S1 and S2; S1 is responsible for receptor binding, whereas S2 (the C-terminal domain) is specifically responsible for viral-cellular membrane fusion [4]. Although SARS-CoV-2 primarily targets the respiratory tract, causing fever, dry cough, sore throat, fatigue, and dyspnoea [5], the virus also results in dysfunction of multiple organ systems outside the lung, including the kidneys, liver, brain, heart, gastrointestinal tract and other organs [6–9], as ACE2 and other candidate receptors are also expressed in these tissues [10].

Emerging studies have revealed the neuroinvasive potential of SARS-CoV-2 [11, 12], with neurological manifestations ranging from lethargy, headache, loss of smell and taste, delirium, insomnia, brain inflammation, stroke, brain haemorrhage to cognitive impairment [13–15]. In fact, the central nervous system (CNS) complications have been observed in more than 30% of individuals with COVID-19 presenting with a higher infection severity [14]. Therefore, studies designed to provide insights into the invasion and effects of SARS-CoV-2 on the CNS are critical. Various studies using cultured cells, animal models, and brain tissues from patients who died of COVID-19, have independently revealed the capacity of SARS-CoV-2 to invade the CNS [16, 17]. Supporting evidence from a postmortem study indicated the presence of viral RNA and proteins in the brains of more than half (21 of 40) of German patients who died of COVID-19 [17], and this phenomenon was confirmed by other neuropathological case studies [18, 19]. In addition, the presence of SARS-CoV-2 in the cerebrospinal fluid (CSF) of infected individuals also confirms CNS infection [15, 20, 21]. Notably, by employing well-characterized human brain organoids, researchers have visualized widespread infectivity of SARS-CoV-2 and extensive death of virus-infected and nearby neuronal cells, and found that the viral infection can be abrogated by pretreatment with an ACE2 antibody or administration of CSF obtained from patients with COVID-19 [12]. Moreover, brain imaging studies have revealed the presence of multiple haemorrhagic lesions and changes in the brain structure of patients infected with SARS-CoV-2, even in milder cases [22–24]. Together, this evidence implies that SARS-CoV-2 has the ability to enter the CNS and cause neurological conditions.

Researchers have speculated that the CNS invasion may occur along nerves or through haematogenous spread [25]. CNS infection may lead to neuroinflammation, blood–brain barrier (BBB) disruption, and alterations in neurovascular and cognition functions, which are closely related to the risk and progression of neurodegenerative diseases [26]. Elderly individuals and those with comorbid conditions are more vulnerable to infection and may be subject to severe outcomes after SARS-CoV-2 infection. Individuals with dementia are three-fold more likely to contract severe COVID-19 condition (requiring hospitalization) than those without dementia [27, 28], and the mortality rate is 30% higher in patients who suffer dementia [29]. Alzheimer’s disease (AD) accounts for more than 60% of all dementia cases [30]. AD pathology is complex and determined by age, heredity, and environmental factors [31]. The main pathological manifestations of AD are the formation of extracellular amyloid-beta (Aβ) plaques and neurofibrillary tangles comprising abnormal tau, as well as neuroinflammation in the brain [32]. The morbidity and mortality rates for COVID-19 are increased in subjects with AD, which is possibly due to the AD-related pathological changes and factors, such as elevated proinflammatory molecules, an advanced age, BBB disruption, presence of *APOE* epsilon4 (*APOE* ε4) allele, diabetes mellitus, and lifestyle factors [33, 34]. Therefore, studies on the associations between AD and SARS-CoV-2 and the potential mechanisms are urgently needed. Here, we summarize the potential routes by which SARS-CoV-2 invades the CNS and then provide an overview of the associations and potential shared pathogenic mechanisms between SARS-CoV-2 and AD, with a focus on the ACE2 receptor, *APOE* genotype, age, and neuroinflammation, in order to advance the understanding of associations between COVID-19 and AD.

## Potential routes of SARS-CoV-2 invasion into the CNS

The presence of viral RNA, proteins or particles in postmortem brain tissues and in the CSF of infected patients indicates the infiltration of SARS-CoV-2 in the CNS. Studies on the mechanisms by which SARS-CoV-2 invades the CNS, which are important for disease diagnosis, prognosis and interventional strategies, are ongoing. Researchers have proposed that SARS-CoV-2 enters the CNS mainly via the direct neuronal route or through haematogenous transport [35, 36].

**Fig. 1:**
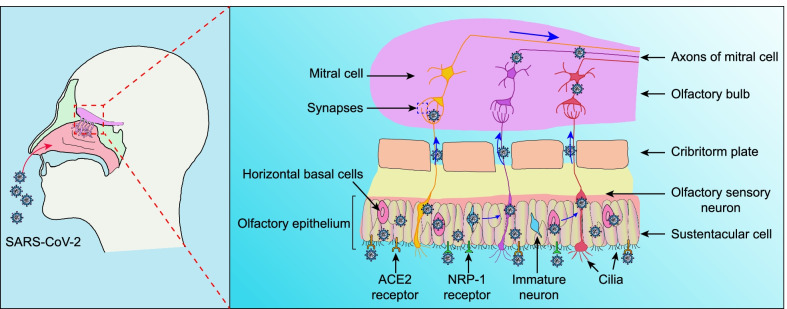
SARS-CoV-2 invades the CNS through the olfactory nerve. Once SARS-CoV-2 enters the nasal cavity, it contacts with olfactory epithelial cells, which express ACE2 and NRP-1 receptors at high levels, rendering the epithelium vulnerable to SARS-CoV-2 infection. The infected olfactory epithelial cells potentially transmit the virus to the surrounding cells, such as horizontal basal cells as well as immature and mature olfactory neurons. The infected horizontal basal cells also mature into olfactory sensory neurons. These neuronal cells extend to the apex, contact the air, and form small nerve bundles at the base before passing through the ethmoid plate and forming the olfactory nerve. Infected olfactory neurons are connected to neurons in the olfactory bulb through synapses, which allows the virus to spread from the olfactory nerve to the olfactory bulb via retrograde transport along axons. The olfactory bulb makes many connections throughout the brain, allowing the virus to spread quickly to other structures in the brain

The neuronal route relies on retrograde axonal transport of the virus from infected peripheral nerves. Evidence shows that SARS-CoV-2 potentially infects the CNS by travelling along olfactory axon bundles. The upper and rear part of the nasal cavity is the olfactory mucosa. The respiratory tract makes direct contact with the CNS through the olfactory sensory neurons. The cilia of the olfactory sensory neurons are present in the nasal cavity, and their axons extend into the olfactory bulb by passing through the ethmoid plate. Intact SARS-CoV-2 particles and viral RNA have been detected in the olfactory mucosa and in neuroanatomical regions that receive olfactory tract projections, indicating that the neuroinvasion occurs via the olfactory pathway [37]. SARS-CoV-2 has been observed to invade the CNS through this route in SARS-CoV-2-infected K18-hACE2 transgenic mice [38] and hamsters [39] (which express the human ACE2 receptor driven by the promoter of the human cytokeratin 18 gene), resulting in a life-threatening disease similar to severe SARS-CoV-2 infection [16, 40]. In those studies, viral RNA was detected in the brains of all animals five days after intranasal administration of SARS-CoV-2, and the severity of neuropathological deficits correlated well with the virus level in the brain. Consistently, in rhesus monkeys exposed to SARS-CoV-2 through intranasal inoculation, the viral RNA copies were sequentially detected in nasal mucosa, olfactory trigone, and entorhinal area, and the viral protein was detected in some functional brain areas, including the thalamus, entorhinal area, medulla oblongata, hippocampus and prepyriform cortex [41]. These findings suggest that SARS-CoV-2 may be transported to the CNS via the olfactory route. In fact, human coronavirus OC43, Middle East respiratory syndrome coronavirus, and SARS-CoV-1 have all been shown to infect the murine CNS via the reverse axonal transport route, invading olfactory sensory neurons in the nasal cavity and then being transported to other neural cells [42–45]. Given the similarities of the viral nucleic acid sequences of coronaviruses [46], SARS-CoV-2 may utilize a similar neural invasion mechanism. The persistence of anosmia and ageusia, typical symptoms of SARS-CoV-2 infection of the olfactory system, in a portion of patients even after recovery from infection, also supports this route of transport. Interestingly, olfactory dysfunction and structural alterations in the olfactory-related regions are early manifestations of several neurodegenerative diseases, such as AD and Parkinson's disease [47–49]. Some researchers have considered that the olfactory nerve is not likely the primary route through which SARS-CoV-2 accesses the brain [50], as human olfactory sensory neurons do not express or exhibit low expression of *TMPRSS2* and *ACE2*, two key genes involved in SARS-CoV-2 entry [51]. Instead, proteins of the two genes are abundant in samples of the whole olfactory mucosa of humans [51] and in mouse olfactory epithelium [52]. Infection of the olfactory epithelium allows the virus to spread to horizontal basal cells as well as immature and mature olfactory neurons. In addition, the infected horizontal basal cells mature into olfactory sensory neurons [53], allowing the virus to reach the olfactory bulb through synaptic connections, thus providing the possibility of CNS infection (Fig. 1). Notably, in addition to ACE2, other receptors such as neuropilin-1 (NRP-1), a signalling protein, have also been discovered to serve as important SARS-CoV-2 receptors and enhance the viral infectivity [54, 55]. NRP-1 is expressed at high levels in the olfactory epithelium, olfactory tubules and paraolfactory gyri, further supporting its role in virus entry into olfactory epithelial cells [54, 56]. These studies indicate that the sustentacular cells are vulnerable to coronavirus entry and give rise to anosmia. Therefore, the olfactory pathway may constitute an important route of CNS invasion. In addition to the olfactory nerve, other peripheral nerves, including the vagus, trigeminus, and nasopharyngeal nerves, may be potential routes through which SARS-CoV-2 accesses the brain [57].

**Fig. 2:**
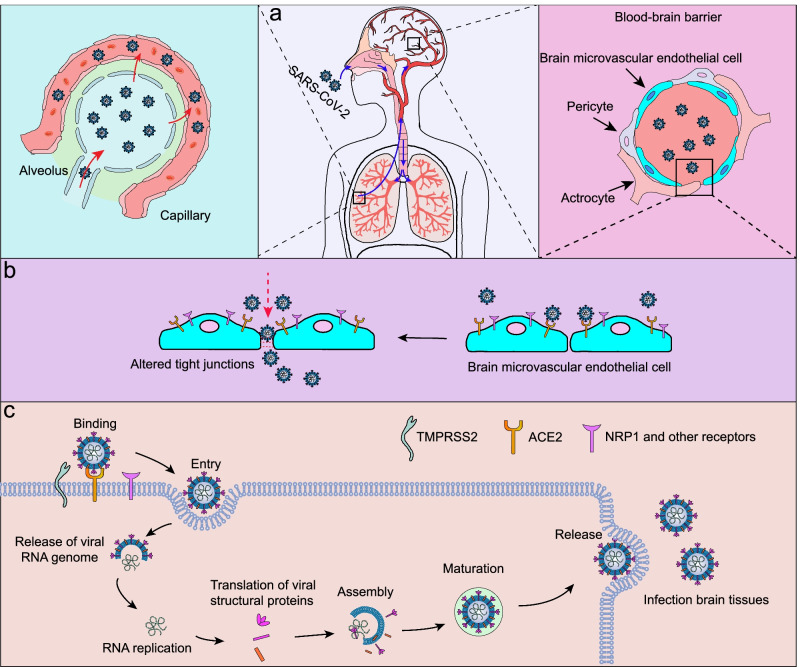
SARS-CoV-2 infects the brain via the haematogenous route. **a** SARS-CoV-2 is a respiratory virus that spreads primarily through airborne droplets, causing infection of the lungs. Left panel: SARS-CoV-2 infection of the lungs may lead to endothelial damage and increased capillary permeability, which allow the transfer of SARS-CoV-2 from the lungs to the pulmonary microcirculation. Right panel: SARS-CoV-2 in the blood can enter the cerebral circulation, where the slow blood flow may allow the virus to damage the BBB. **b** Viruses in the cerebral circulation may infect and destroy microvascular endothelial cells, resulting in increased BBB permeability and facilitating viral entry into brain tissues. **c** SARS-CoV-2 infects brain cells via the interaction of S protein with ACE2, NRP1 and other potential receptors on microvascular endothelial cells after the S protein is primed by TMPRSS2. Once SARS-CoV-2 enters the cell, the viral life cycle begins, including genome replication, protein synthesis, virus assembly, maturation and release, ultimately leading to brain parenchyma infection and tissue damage

In addition to the olfactory route, SARS-CoV-2 may also enter the CNS via the haematogenous route; however, in this route the virus must be able to cross the BBB to achieve entry into the CNS. The BBB, which is composed of endothelial cells, pericytes, and astrocytes, functions as an important supporting and protective interface that restricts the entry of circulating molecules, such as neurotoxic plasma components, leucocytes, and pathogens, into the brain [58]. SARS-CoV-2 infection of the lungs may lead to endothelial damage and increased capillary permeability [59, 60], which facilitates virus transfer from the lungs to the pulmonary microcirculation (Fig. 2a). SARS-CoV-2 in the blood will enter the cerebral circulation, where the slow blood flow may promote potential interactions of S protein with ACE2 [61] and NRP-1[62] on microvascular endothelial cells [35]. The presence of virus-like particles in the brain capillary endothelium in the frontal lobe of a patient who died from COVID-19 provides direct evidence for viral invasion into brain microvascular endothelial cells of the BBB [63]. Autopsy studies have revealed that SARS-CoV-2 disrupts the BBB. This has been confirmed in 2D static and 3D microfluidic engineered models of the human BBB [64]. In this model, the SARS-CoV-2 S protein activates the proinflammatory response in the brain endothelium and contributes to increased barrier permeability, which facilitates viral entry into the brain (Fig. 2b). In addition, viral RNA occasionally appears in the vascular wall, perivascular region, and brain microvascular endothelium of SARS-CoV-2-challenged K18-hACE2 transgenic mice [65]. Other mechanisms by which SARS-CoV-2 affects the BBB integrity include invasion of BBB epithelial cells and astrocytes, disruption of placodes or the actin cytoskeleton, and phosphorylation of tight junction proteins [66, 67]. SARS-CoV-2 also infects leukocytes and enters the BBB via these cells [68, 69]. Systemic virus dissemination in the CNS may also occur via exosomal cellular transport and lymphatic spread following infection, immune activation, and production of granulocyte macrophages [70]. Recently, researchers have observed that challenge with SARS-CoV-2 results in infection of the epithelium of the choroid plexus and leads to the breakdown of the blood-CSF barrier in an organoid model of the human choroid plexus, providing another entry route for SARS-CoV-2 into the brain [71]. Ultimately, the virus in the CSF must infect or penetrate the BBB to enter the brain. Notably, AD in particular has an early pathology associated with increased permeability and disruption of the BBB, which, together with reduced cerebral blood flow, can place individuals at a higher risk of contracting SARS-CoV-2 [72–74]. Moreover, even older individuals who are not yet symptomatic for AD (but who are perhaps very early in the pathological trajectory in developing AD) may experience some BBB degeneration and an early loss of pericytes, which promote increased vulnerability to the virus. After the virus enters brain cells, the viral life cycle begins, including genome replication, protein synthesis, assembly, maturation and virus release, ultimately leading to brain tissue infection and neurological symptoms (Fig. 2c). It has been widely demonstrated that vaccines are the most efficient tools for preventing COVID-19. Studies have reported that COVID-19 vaccines are safe and well-tolerated in patients with neurological disorders [75]. Nevertheless, a recent study reported that vaccinated patients with AD displayed a significantly increased overall risk for breakthrough infections (approximately 10.3%) than the matched older adults without dementia (approximately 5.6%) [76]. However, after further matching for comorbidities, such as heart diseases, cancers, type 2 diabetes, and hypertension, dementia patients no longer showed an obvious increase in risk of breakthrough infections. In addition, several rare neurological complications can occur in individuals who received COVID-19 vaccines (ChAdOx1nCoV-19 and BNT162b2), such as Guillain–Barré syndrome, Bell’s palsy and myasthenic disorders [77], whereas such complications are much more common among individuals with SARS-CoV-2 infections. Future studies are warranted to evaluate the possible use of vaccines in preventing the spread of viral infection throughout the CNS, as well as to evaluate the protective effects of vaccination on AD and the potential side effects.

In conclusion, there are two major potential routes by which SARS-CoV-2 invades the brain: the olfactory pathway and haematogenous transport. Despite the evidence presented here, the primary invasion route, the mechanism by which SARS-CoV-2 spreads throughout the brain, and the mechanisms of SARS-CoV-2-induced damage to the CNS remain unclear and require further studies. Furthermore, how the coronavirus enters the bloodstream remains an open question.

## Common risk factors and mechanisms of COVID-19 and AD

### ACE2: a possible double-edged sword in AD

The transmembrane glycoprotein ACE2 is a vital component of the renin-angiotensin system (RAS) that is responsible for catalysing the conversion of angiotensin (Ang) II into Ang (1–7), thus protecting against the harmful effects of the ACE1/Ang-II axis [78]. Ang-II exerts vasoconstrictive, proinflammatory hypertrophy, and profibrotic effects, while Ang (1–7) exerts opposing effects, such as antiproliferative, anti-inflammatory, antiapoptotic, and mild vasodilatory effects, to protect against various cardiovascular diseases [79, 80]. In addition to acting as a negative regulator of RAS, ACE2 also plays a protective role in inflammatory lung diseases in other manners. Studies have identified that des-Arg^9^ bradykinin (DABK) is a biological substrate of ACE2 in airway epithelial cells, and the attenuation of ACE2 activity facilitates neutrophil infiltration, which is partially attributed to the modulation of DABK/bradykinin receptor B1 axis signalling [81]. Since the onset of the COVID-19 pandemic, ACE2 has been recognized as the main host cell receptor for SARS-CoV-2 infection. The amount and distribution of ACE2 may influence the risk of SARS-CoV-2 infection. As the respiratory tract is the dominant target of SARS-CoV-2, high levels of ACE2 have been detected in respiratory epithelial cells and pulmonary type II alveolar cells [82]. However, ACE2 expression is not restricted to the respiratory tract, as kidney proximal tubule cells, bladder urothelial cells, ileum and oesophageal epithelial cells, neurons and glial cells in the brain, and myocardial cells all exhibit high levels of ACE2 [61, 83–87]. As a result, the function and hyperactivity of the ACE2 receptor may increase the sensitivity of these target cells and organs to SARS-CoV-2 infection. ACE2 plays a key role in SARS-CoV-2 neuroinfection, but its expression in the context of AD remains controversial. A recent study reported that the ACE2 protein level is upregulated in the hippocampal tissues of patients with AD and that the change is not age- or sex-dependent, indicating a direct relationship between AD and ACE2 expression [88]. Another study detected ACE2 expression in multiple brain regions of a recently deceased patient with AD, and ACE2 expression was significantly increased in the temporal lobe and CA1 region of the hippocampus [89]. In addition, RNA-seq showed that the expression of ACE2 mRNA is elevated in cortical tissues from  5× FAD mice [90] but not significantly altered in the blood. However, previous studies have reported inconsistent findings for ACE2 expression and its effect on AD pathology. Immunohistochemical staining showed that ACE2 expression varies across different brain regions, decreased in the hippocampus, visual cortex, basal nucleus, amygdala, middle frontal gyrus and entorhinal cortex in response to AD pathology [91]. Specifically, ACE2 activity is obviously decreased in the postmortem brain tissues of patients with AD compared to those of age-matched controls, and is inversely related to Aβ levels and tau phosphorylation [92]. The decrease of ACE2 expression in the context of AD indicates the occurrence of RAS dysregulation and neurological damage in corresponding brain regions. Additional studies have also supported this hypothesis. For instance, elevated ACE2 activity in the brain induced by intraperitoneal administration of an established ACE2 activator (diminazene aceturate) reduces Aβ-related pathology and delays cognitive impairment in symptomatic (aged) Tg2576 mice and protects against the onset of cognitive decline in presymptomatic (younger) Tg2576 mice [93]. A previous study also found that a longer, neurotoxic species of Aβ (Aβ43) is converted into a shorter, less toxic or neuroprotective form of Aβ (Aβ40) by ACE and ACE2 [94] and that this conversion is reversed by the specific ACE2 inhibitor DX600. The researchers also found that ACE2 activity is decreased in the sera of patients with AD compared with the sera of age-matched controls, indicating a relationship between lower ACE2 activity and AD. Likewise, in a *D*-galactose-ovariectomized rat model of ageing and dementia, increased activation of ACE2 via administration of dimenazine ameliorates Aβ pathology in the brain and improves cognitive performance [95]. Therefore, during the initial stages of AD, maintaining ACE2 activity in the brain may be a protective factor, limiting the progression of AD pathology. Thus, researchers have speculated that the SARS-CoV-2 infection-induced downregulation of ACE2 expression in target cells [96] may inhibit the protective effect of ACE2 against AD. However, ACE2 levels in the CSF are similar between patients with AD and controls [97]. Furthermore, no significant difference in ACE2 activity was observed in the cerebral cortex or hippocampus of SAMP8 mice, a non-transgenic animal model of sporadic AD, during disease progression [98]. Possible explanations for the inconsistency in results among these studies might be the differences in animal models used, brain areas selected, and detection methods employed.

In conclusion, high levels of ACE2 enhance SARS-CoV-2 infection, whereas ACE2 expression decreases once viral infection occurs, and the protective function of ACE2 is inhibited. At present, the changes in ACE2 expression that occur in the context of AD are controversial. Future studies using more animal models and larger sample sizes, especially specimens from more patients with AD, will help to clarify the expression of ACE2 in individuals with AD and the relationship between ACE2 and AD pathology. Additionally, the role of ACE2 in virus transmission throughout the brain requires further study.

### APOE4: increased susceptibility to COVID-19

The manifestations of SARS-CoV-2 infection vary widely among individuals, from no symptoms to life-threatening conditions, suggesting that the host genetic background may influence the risk and severity of virus infection [99, 100]. An important genetic factor is the *APOE* genotype. ApoE is a multifaceted secreted protein synthesized by astrocytes in the CNS and by the liver in the periphery [101]. Human *APOE* encodes a 299-amino-acid protein with three major isoforms that differ at amino acid residues 112 and 158, i.e., apoE2 (Cys^112^, Cys^158^), apoE3 (Cys^112^, Arg^158^), and apoE4 (Arg^112^, Arg^158^). The amino acid differences exert profound effects on the apoE protein structure and function [102, 103]. *APOE*4 is the strongest genetic susceptibility factor for sporadic AD [104], accounting for more than 95% of all AD cases [105]. ApoE2 is protective, and apoE3 is neutral [106]. Previous studies have reported that *APOE*4 is related to the occurrence and severity of infections with viruses and pathogenic microorganisms, such as HIV-1 [107], herpes simplex [108] and hepatitis C [109]. Since the onset of the COVID-19 pandemic, an increasing number of studies have been conducted on the possible associations of *APOE* polymorphisms with SARS-CoV-2 infection and disease severity. Recently, Kuo et al. found that *APOE* ε4/ε4 homozygotes exhibit a more than two-fold higher susceptibility to COVID-19 [110, 111] and a four-fold higher risk of mortality than *APOE* ε3/ε3 homozygotes, according to data from the UK Biobank cohort [110, 111]. The association was not diminished even after controlling for *APOE*4-related diseases, including coronary artery disease, hypertension, diabetes, and dementia, suggesting that *APOE*4 may exert an independent effect on COVID-19 severity. Other studies have reported similar findings in Spanish and Iraqi subjects: the ε4 allele of the *APOE* gene increases the incidence and clinical severity of SARS-CoV-2 infection [112, 113]. Another study showed no obvious difference in the frequency of the ε4 allele between viral RNA-positive subjects and the control population in a Czech cohort [114]. When the data were further analysed after stratification according to the disease status, the results revealed a significantly higher frequency of the ε4 allele in symptomatic subjects, indicating that the ε4 allele may be associated with an increased risk of symptomatic COVID-19. Notably, a recent study on a Finnish population indicated that the ε4 carriers are prone to severe COVID-19 and prolonged mental fatigue, partially due to cerebrovascular damage [115]. The effect of *APOE*4 on the susceptibility to and the severity of COVID-19 may provide an explanation for the variations of severity of COVID-19 among different ethnic groups. The frequency of the ε4 allele is approximately 30%–40% in black Africans and approximately 7%–20% in Caucasians [116]. Studies have indicated that the burden and the mortality rate of COVID-19 are disproportionately higher in black persons/African Americans than in white persons [117, 118], and black individuals with dementia are more prone to contracting SARS-CoV-2 than white individuals with dementia [119]. In contrast, Gunanidhi D et al. [120] conducted a global epidemiological study on the correlation of apoE isoforms with COVID-19 morbidity and mortality and observed a significant but negative relationship between the ε4 allele and COVID-19 susceptibility. When the data were further analysed after stratification according to ethnicity, the researchers observed that the ε4 isoform protected against COVID-19 in Asians but not in Europeans, Africans or Americans.

**Fig. 3:**
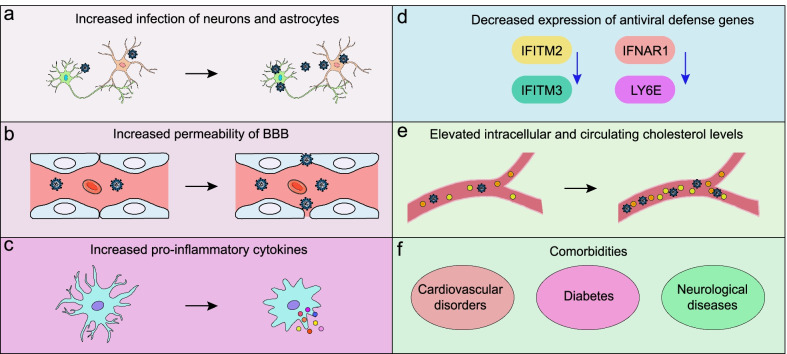
*APOE*4 contributes to increased susceptibility to COVID-19. **a** *APOE*4 was shown to increase the infectivity of SARS-CoV-2 in iPSC-derived neurons and astrocytes. **b** *APOE*4 has been reported to be associated with increased BBB permeability. **c** ApoE4 is related to increased production of proinflammatory cytokines by macrophages in the periphery and by microglial cells in the CNS. **d** Compared to AD patients presenting the *APOE* ε3/ε3 genotype, AD patients with the ε4/ε4 genotype display decreased expression of several antiviral defence genes, such as *IFITM2*, *IFITM3*, *IFNAR1* and *LY6E.* **e** Individuals carrying the ε4 allele exhibit elevated intracellular and circulating cholesterol levels, and a high level of cellular cholesterol may facilitate SARS-CoV-2 infection. **f** *APOE*4 is a risk factor for several cardiovascular and neurological diseases, and individuals with these pre-existing comorbidities are more vulnerable to infection and poor clinical outcomes. Thus, individuals with the *APOE*4 genotype are more susceptible to SARS-CoV-2 infection, which may lead to neurodegeneration

Multiple molecular mechanisms may underlie the possible relationship of *APOE* polymorphisms with SARS-CoV-2 infection and COVID-19 severity, and some of the mechanisms remain speculative. Recently, an in vitro study revealed that the *APOE*4 genotype increases the rate of SARS-CoV-2 infection of human induced pluripotent stem cell (iPSC)-derived neurons and astrocytes, and that *APOE*4-expressing astrocytes produce a detrimental response compared with *APOE*3 carriers [26] (Fig. 3a). These results suggest that *APOE*4 may exert a causal effect on the severity of COVID-19. Another potential mechanism is that *APOE*4 is associated with increased BBB permeability [121], which allows the virus to enter the CNS more easily through BBB leakage and makes patients more susceptible to infection [122] (Fig. 3b). Moreover, *APOE*4 has been reported to promote production of proinflammatory cytokines (such as IL-6 and TNF-α) by macrophages in the CNS and the periphery in response to proinflammatory stimuli [123–125] (Fig. 3c). As the cytokine storm has been identified as a critical hallmark of severe COVID-19 [126], *APOE*4 may be responsible for the poor outcomes of patients with COVID-19 due to its effect on the host immune response. Additionally, compared to AD patients with the *APOE* ε3/ε3 genotype, those with the ε4/ε4 genotype exhibit decreased expression of several antiviral defence genes, such as *IFITM2*, *IFITM3*, *IFNAR1* and *LY6E* [127] (Fig. 3d). Compared to the two other isoforms, *APOE*4 is genetically associated with reduced apoE levels, which increases the risk of coronavirus infection and disease progression, and this reduction is consistently associated with severe COVID-19 [128]. Notably, *APOE*4 may contribute to the infectivity of SARS-CoV-2 by regulating intracellular cholesterol levels. ApoE is an amphipathic protein that is responsible for regulating organismal lipid and cholesterol homeostasis [129]. Individuals carrying the ε4 allele exhibit elevated intracellular and circulating cholesterol levels [130], and a high cellular cholesterol level may facilitate SARS-CoV-2 infectivity by increasing the binding of the S protein to the ACE2 receptor [131, 132] (Fig. 3e). Moreover, a recent molecular docking study revealed that the receptor-binding domain of SARS-CoV-2 binds to the apoE protein, potentially leading to the exposure of the active site of apoE; thus, the virus may enter host cells via apoE-related metabolic pathways [133]. However, this interaction must be confirmed by experimental studies, and whether this binding effect depends on the apoE isoform remains to be determined. Additionally, the interactions between apoE and the ACE2 receptor should be investigated to determine the direct relationship between *APOE* and COVID-19. Pathologically, apoE has been shown to be coexpressed with ACE2 in alveolar epithelial cells [134], which are the primary targets of SARS-CoV-2, and treatment with rhACE2 or genetic ablation of ACE2 has been shown to modulate apoE-related physiological functions [135]. However, the correlation between apoE and ACE2 levels should be examined in the context of COVID-19. Additionally, *APOE*4 is a risk factor for several cardiovascular and neurological diseases, such as AD [102], atherosclerosis [136], and diabetes [137]. Individuals with these pre-existing comorbidities are more vulnerable to developing severe COVID-19 and having higher mortality rates following SARS-CoV-2 infection [138, 139] (Fig. 3f). Thus, although apoE may exert independent effects on the risk of infection and disease severity, these comorbidities should be considered.

In summary, the inconsistency in the findings related to the association between *APOE* gene polymorphism and COVID-19 risk and severity may be due to the differences in the genetic characteristics and age of the included subjects, the case definitions and the number of subjects employed. Further research in a larger sample size and employing subjects with different ethnic backgrounds will help identify those who are at high risks of infection and severe disease, in order to help prioritize health care and preventive measures. In addition, the direct effects of apoE isoforms on COVID-19 severity and the related pathways require further investigations in patients, animals, and cells to better delineate the major underlying mechanisms, thus facilitating the development of targeted interventions.

### Age: a primary common risk factor for COVID-19 and AD

Age is the greatest risk factor for AD [140], as the prevalence and mortality of AD increase substantially with age [141]. Approximately one in 10 people aged ≥ 65 years has AD, and the prevalence increases to 32% in those aged ≥ 85 years, among whom the per-year incidence rate is approximately 6.5% [142]. Age affects the pathology of AD in a number of ways, such as by causing dysregulation of innate immune responses, impairment of the BBB, and alterations in neuroinflammation [140]. Accumulating evidence has shown that elderly individuals, especially those with comorbidities such as chronic obstructive pulmonary disease, hypertension, cancers, and diabetes, are particularly vulnerable to SARS-CoV-2 infection and are more likely to develop severe illness [143–145]. Notably, a systematic study assessed the clinical, molecular, and immunological characteristics of 326 patients with COVID-19 from China and found that age, among other factors, is the most obviously associated with disease severity [146]. Early evidence from China indicated that the case-fatality ratio (CFR) of COVID-19 is less than 0.4% among patients aged 40 years and younger, 8% among patients aged 70–79 years and a startling 14.8% among those aged 80 years and older [147]. A consistent and more pronounced age-related trend in the CFR of COVID-19 has been reported in the Italian population: patients aged 40 years or younger have a CFR of less than 0.4%, whereas the CFR is markedly increased to 12.8% in patients aged 70–79 years and further increased to 20.2% in those aged ≥ 80 years [148]. In addition, the overall CFR in the Italian population (7.2%) was reported to be substantially higher than that observed in China (2.3%), possibly in part because that Italy has one of the highest percentages of elderly individuals worldwide, with an average life expectancy of 82.3 years and a proportion of 23% for individuals aged 65 years or older. Similar exponential increases in the CFR of COVID-19 with age have been reported in other countries. A meta-analysis of more than 600,000 patients with COVID-19 from the United Kingdom, China, Spain, Italy, and New York State reported that the mortality rate in patients aged 60–69 years was more than three times higher than that in those aged 50 to 59 years [149].

Several potential factors may account for the marked increase in the risk of SARS-CoV-2 infection and poor clinical outcomes in the elderly. First, age-related impairment and dysregulation of the host innate and adaptive immune systems may lead to decreased protection against virus infection and result in a state of chronic inflammation [150–152]. In addition, the levels of inflammatory markers, such as C-reactive protein (CRP), Toll-like receptor (TLR), TNF-α, IL-6, and IL-1β, generally increase with age [153, 154]. These changes result in the low-grade proinflammatory state observed in older populations [155], which may contribute to severe disease conditions. Specifically, increased serum CRP levels, which indicate an inflammatory state and are associated with advanced age, indicate a poor prognosis and increased risk of mortality in elderly patients with COVID-19 [156, 157]. Additionally, serum CRP levels have been used to monitor COVID-19 progression and patients’ response to COVID-19 treatments, such as tocilizumab [158]. Notably, loss of cerebrovascular integrity, a feature of ageing, may enable pathogens to enter the brains of older individuals [159]. Moreover, increases in the expression of ACE2, the major receptor for SARS-CoV-2, with age in several human tissues, such as the nasal epithelium [160], lung [145], and kidney [161], may increase the risk of S protein binding and the development of severe COVID-19 in older adults; however, this speculation remains controversial. Furthermore, intracellular cholesterol accumulates in type-2 pneumocytes and alveolar macrophages with ageing [162, 163]. As described above, a high cellular cholesterol level may facilitate SARS-CoV-2 infection by enhancing the S protein-ACE2 interaction [131, 132], consistent with the increase in poor COVID-19 outcomes in the aged population. Notably, age-related comorbidities, such as hypertension, cardiovascular disease, dementia, diabetes mellitus, kidney failure, cancer and metabolic syndrome, become more prevalent with ageing, and each of those pre-existing comorbidities increases the susceptibility to poor clinical outcomes after SARS-CoV-2 infection [164, 165]. In particular, memory impairment may affect the ability of elderly patients to comply with COVID-19 precautions, such as mask wearing, hand hygiene, and maintaining appropriate social distance. Together, these potential factors may individually or collectively render older individuals more vulnerable to coronavirus infection and lead to severe disease.

Age is clearly a major risk factor for COVID-19, possibly due to the age-related dysfunction of the immune system, inflammation and comorbidities. However, the molecular mechanisms of age-related increases in disease vulnerability and severity are currently poorly understood, which would be an important area of investigation.

### Neuroinflammation: an important bridge between COVID-19 and AD

**Fig. 4:**
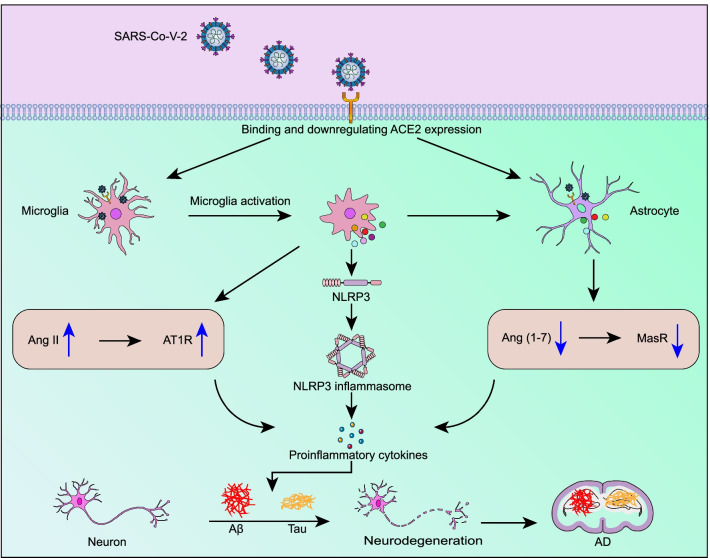
SARS-CoV-2 infection causes neurodegeneration by triggering neuroinflammation. SARS-CoV-2 infection causes activation of microglia and astrocytes, triggering a neuroinflammatory cascade. Activated microglia secrete inflammatory cytokines, which promote astrocyte activation and NLRP3 inflammasome formation. Binding of SARS-CoV-2 to ACE2 downregulates ACE2 expression, which may increase Ang II expression and subsequently activate AT1R in microglia, resulting in NF-κB expression and a proinflammatory response. On the other hand, downregulation of ACE2 activity is associated with decreased Ang (1–7) levels and subsequent MasR activity in astrocytes, which may lead to the production of ROS and proinflammatory factors in the CNS parenchyma and cerebral vessels. These processes result in Aβ deposition and tau phosphorylation, which ultimately lead to neurodegeneration and AD

Neuroinflammation is recognized as another characteristic pathophysiology of AD [166]. Microglia and astrocytes are major sources of cytokines in individuals with AD [167]. Dysfunction of the immune system may promote the release of proinflammatory cytokines and result in synaptic damage, neuronal death, and inhibition of neurogenesis, which are related to the pathogenesis of AD [168]. Although accumulating evidence indicates that SARS-CoV-2 can enter the CNS, the presence of the virus in the brain may not critically correlate with the neurological conditions, as postmortem studies have noted occurrence of pronounced neuropathological changes even in patients with COVID-19 in whom the virus was not detected in the CNS [17, 19, 37, 169, 170]. Instead, extensive research on CSF and postmortem brain tissues has indicated that immune dysfunction and pronounced neuroinflammation within the CNS are the main driver of CNS damage and neurological symptoms in infected individuals. Neuroinflammation might result directly from coronavirus infection or from excessive peripheral inflammation. Both SARS-CoV-2 and proinflammatory mediators may promote BBB disruption, allowing the virus to cross the BBB, enter the CNS [171], and activate microglia and astrocytes, triggering a neuroinflammatory cascade that may contribute to the onset and progression of neurodegeneration [172–174] (Fig. 4).

Under physiological conditions, microglia act as resident phagocytes in the CNS parenchyma, which are critical for homeostasis, facilitate synaptogenesis, regulate synaptic pruning and myelination, and subsequently support neuronal survival [175, 176]. Under pathological conditions, microglia rapidly respond to CNS pathogens or excessive peripheral inflammation by expressing specific surface receptors, driving morphological changes, and producing proinflammatory molecules and reactive oxygen species (ROS), which exacerbate neuroinflammation [177]. Microglia are likely to be affected by SARS-CoV-2 entry into the brain, as NRP-1, an important receptor for the virus, has recently been shown to be expressed in microglia [54]. Several previous studies have suggested that the S protein of SARS-CoV-2 might function as a pathogen-associated molecular pattern (PAMP) to drive neuroinflammatory responses through activation of TLRs, which are widely expressed on macrophages and microglia and may function as pattern recognition receptors (PRRs) to recognize molecular sequences common to bacterial and viral pathogens [178–180]. Microglia have been shown to express a wide range of PRRs, including TLRs, NOD-like receptors, RAGE and scavenger receptors [181]. SARS-CoV-2 activates these receptors, possibly eliciting neuroinflammatory responses and contributing to disease progression and severity. Widespread microglial activation, which may lead to activation of lymphocytes and T cells [17], has been observed in brains of the majority of patients with COVID-19 [182]. SARS-CoV-2 infection-induced activation of microglia may increase the expression of TNF-α, IL-6, IL-1β, nitric oxide, and chemokine C–C motif ligand 2, which may further increase the AD-related tau pathology [183, 184]. In addition to being potential targets of coronaviruses, microglia are also highly susceptible to the effects of proinflammatory mediators. Studies have shown that microglial activation, microglial nodules and neuronophagia are present in postmortem brain tissues of the majority of patients with COVID-19 and that these changes are not directly due to SARS-CoV-2 infection but likely result from systemic inflammation [185], as the viral RNA or protein is not detected in the microglial nodules or neuronophagic cells.

Astrocytes are also important components of the intrinsic immune response in the CNS following viral infection. An increased plasma concentration of glial fibrillary acidic protein (GFAP), a hallmark of astrocyte activation, is commonly detected in patients with moderate and severe COVID-19 [186]. As components of the BBB, astrocytes are highly sensitive to peripheral inflammation. In addition to responding to signals from microglia, astrocytes also rapidly respond to proinflammatory cytokines secreted by endothelial cells. Astrocytes express inflammatory molecules that contribute to neuroinflammation and neurodegeneration upon exposure to activated proinflammatory microglia, including proinflammatory cytokines such as IL-6, IL-1β and TNF-α; PAMPs; and danger-associated molecular patterns [187]. These data clearly indicate that microglia and astrocytes participate in neuroinflammatory responses to viral infection of the CNS.

In addition, the systemic inflammatory response initiated by SARS-CoV-2 infection is partially mediated by hyperactivation of the NLRP3 inflammasome [188, 189]. Activation of the NLRP3 inflammasome decreases the phagocytosis of Aβ by microglia, thereby increasing the deposition of Aβ and subsequently facilitating the onset and development of AD pathology [190]. Hyperactivity of the intracellular NLRP3 inflammasome facilitates the polarization of microglia towards the M1 phenotype, resulting in Aβ deposition and increased cognitive deficits in transgenic AD mice [191]. Conversely, deficiency of the NLRP3 inflammasome results in the polarization of microglia towards the M2 phenotype, thus reducing the formation of Aβ plaques, inhibiting synaptic injury, and mitigating cognitive decline [192]. Activation of the NLRP3 inflammasome has also been reported to promote tau pathology, which promotes the occurrence and progression of AD [193]. Furthermore, hyperactivation of P2X7 receptors has been reported to be closely associated with the inflammatory process, as P2X7 receptors are stimulated by adenosine triphosphate (ATP) released from damaged cells, inducing inflammasome activation [194]. The increase of extracellular ATP level caused by SARS-CoV-2 infection may trigger hyperactivation of the P2X7 receptor, leading to stimulation of the NLRP3 inflammasome [195]. In this context, the P2X7 receptor may be an appealing target for prevention or treatment of neurological manifestations of COVID-19. In addition, comorbidities, such as obesity, heart disease, diabetes and hypertension, which are related to poor outcomes, are associated with pronounced inflammation in patients with COVID-19 [196].

Additionally, downregulation of ACE2 expression caused by binding of the SARS-CoV-2 S protein promotes cellular and tissue damage, possibly further aggravating neuroinflammation, oxidative stress and cerebrovascular endothelial injury. Oxidative stress and cyclooxygenase (COX)-1- or COX-2-mediated neuroinflammation are substantially inhibited in transgenic mice with neuron-specific overexpression of ACE2, resulting in improvement of the antioxidant status and nitric oxide homeostasis [197]. Therefore, pharmacological activation of ACE2 may exert beneficial effects on the neuropathological sequelae of SARS-CoV-2 infection [198–200]. Moreover, neuroinflammation might result from dysregulation of the RAS in the CNS. As described above, SARS-CoV-2 binding downregulates ACE2 expression, which may increase the expression of Ang II and subsequently activate AT1R in microglia, resulting in promotion of NF-κB expression and proinflammatory responses. On the other hand, decreased ACE2 activity is associated with decreases of Ang (1–7) level and MasR activity in astrocytes, which may lead to the production of ROS and proinflammatory factors in the parenchyma and cerebral vessels of the CNS. Neurodegeneration may subsequently occur. Furthermore, abnormalities in the levels of CSF biomarkers, including increased level of inflammatory mediators, have been observed in patients with COVID-19, suggesting that the increased cytokine and chemokine levels in the CSF following virus infection may contribute to neuroinflammation [201–203].

In addition to being a major contributor to cardiovascular diseases, hypertension has been demonstrated as a risk factor for AD [204]. Ang II receptor blockers (ARBs) are commonly used antihypertensive medications, and they have high affinity for AT1R, antagonizing the interaction between Ang II and its AT1R receptor. Regarding the effects of ARBs on AD, both in vitro and in vivo evidence has shown that treatment with ARBs can ameliorate most of the clinical risk factors of AD [205]. Although several studies failed to show restoration of spatial memory and learning deficiencies by the treatment [206, 207], an increasing number of studies reported that treatment with ARBs, such as losartan, candesartan, telmisartan, olmesartan or valsartan, is beneficial for memory and cognition in vivo [208–210]. In addition, administration of valsartan, losartan  or telmisartan  is capable of lessening the Aβ plaque burden [211, 212]. Evidence regarding the effect of ARBs on phosphorylated tau, another important hallmark of AD, is scarce. It seems that telmisartan is able to decrease the hippocampal content of hyperphosphorylated tau protein and neurofibrillary tangles [213, 214]. In addition to the aforementioned Aβ and tau, ARBs can also reduce neuroinflammation [206, 215]. Notably, a recent study investigated the effects of ARBs and ACE inhibitors (ACEIs) that are prescribed to treat COVID-19 in patients with AD or mild cognitive impairment (MCI). Although there was no significant relationship between ARB or ACEI use and the severity of COVID-19 among AD and MCI patients, the authors found that the ARBs were associated with a reduced risk for COVID-19 [216].

In conclusion, neuroinflammation is caused both directly by SARS-CoV-2 infection of the CNS and indirectly by peripheral inflammation via immune-to-brain signalling. However, further research assessing the driver of the major pathological processes, the duration of neuroinflammation after SARS-CoV-2 infection, and whether this neuroinflammation exerts long-term effects on the nervous system is warranted.

### Other potential factors that mediate the association between COVID-19 and AD

In addition to the connections described above, other factors responsible for the relationship between AD and COVID-19 are being explored. According to a recent study, Aβ42 increases SARS-CoV-2 pseudovirus infection by strengthening the binding of the S1 subunit to the ACE2 receptor, suggesting that Aβ42 may play an important role in the development of severe COVID-19 [217]. Researchers also concluded that SARS-CoV-2 infection aggravates AD by exacerbating neurotoxicity and increasing the levels of Aβ, inflammation and oxidative stress [218, 219]. In addition, SARS-CoV-2-induced neuronal damage in 3D human brain organoids may be attributed to aberrant changes in the distribution and phosphorylation of tau [220]. Consistent with this study, SARS-CoV-2 infection activates biochemical pathways associated with tau pathology, which is one of the major factors that drive AD pathology [221]. Moreover, cognitive impairment and neuropsychiatric symptoms make it difficult for AD patients to understand and follow safety measures [14]. All these factors reinforce the potential link between COVID-19 and AD.

## Conclusions and perspectives

The ongoing global COVID-19 pandemic caused by SARS-CoV-2 has extensively altered our daily lives. Infection is not only restricted to the respiratory system but also affects the CNS and leads to neurological manifestations. The morbidity and mortality rates of COVID-19 are increased in patients with AD. The mechanism by which the virus gains access to the CNS and why patients with AD are at higher risk of virus infection are not well understood. In this review, we summarize the current literature on the possible routes by which SARS-CoV-2 invade the CNS and further analyse the shared aetiological cofactors and potential mechanisms linking COVID-19 and AD, which may advance our understanding of the inherent relationship between the two diseases. Although people infected with SARS-CoV-2 frequently display neurological symptoms, multiple manifestations remain unrecognized. First, neurological symptomatology is not always present, and neurological symptoms are often ignored because the first goal is to combat respiratory symptoms. In addition, clarifying the mechanisms by which SARS-CoV-2 invades and spreads throughout the CNS is important for preventing and ameliorating neurological symptoms; however, the precise mechanism by which SARS-CoV-2 invades the CNS has not been completely elucidated. Moreover, researchers have not clearly determined whether the neuropathological alterations are directly due to the neuroinvasion by the virus or caused indirectly by the dysregulation of the immune system following infection. Emerging studies are focusing on the effect of the coronavirus on the onset and progression of AD. However, as it has not been long since the onset of the COVID-19 pandemic, whether SARS-CoV-2 infection contributes to long-term cognitive dysfunction and behavioural impairments in AD patients and triggers AD in infected individuals remain central questions. Therefore, long-term follow-up cohort studies designed to determine the long-term effects of COVID-19 on AD are urgently needed. Additionally, the relationships of AD-related pathological markers, risk factors and behavioural changes with COVID-19 must be further established. Moreover, as the COVID-19 pandemic continues, new SARS-CoV-2 mutants (e.g., delta and omicron strains) will continue to emerge, and the ability of these mutants to infect the CNS and their effect on AD must be elucidated. In summary, studies are imperatively needed to clarify the potential mechanisms underlying the elevated susceptibility and mortality rate of AD patients to COVID-19 and discover preventive strategies to minimize the risk of viral infection among patients with AD.

## Data Availability

Not applicable.

## Abbreviations

COVID-19: Coronavirus disease 2019
SARS-CoV-2: Severe acute respiratory syndrome coronavirus 2
ACE2: Angiotensin-converting enzyme 2
AD: Alzheimer’s disease
CNS: Central nervous system
APOE: Apolipoprotein E
TMPRSS: Transmembrane serine protease 2
CSF: Cerebrospinal fluid
BBB: Blood–brain barrier
Aβ: Amyloid-beta
NRP-1: Neuropilin-1
RAS: Renin-angiotensin system
Ang: Angiotensin
CFR: Case-fatality ratio
CRP: C-reactive protein
TLR: Toll-like receptor
ROS: Reactive oxygen species
PAMPs: Pathogen-associated molecular patterns
PRRs: Pattern recognition receptors
GFAP: Glial fibrillary acidic protein
COX: Cyclooxygenase
ARBs: Angiotensin II receptor blockers
ACEIs: ACE inhibitors (ACEIs)
MCI: Mild cognitive impairment
